# Accuracy of a point-of-care blood lactate measurement device in a prehospital setting

**DOI:** 10.1007/s10877-022-00812-6

**Published:** 2022-01-27

**Authors:** Louise Houlberg Walther, Floor Zegers, Mads Nybo, Christian Backer Mogensen, Erika Frischknecht Christensen, Annmarie Touborg Lassen, Søren Mikkelsen

**Affiliations:** 1 The Prehospital Research Unit, Region of Southern Denmark, Odense University Hospital, Odense, Denmark; 2 Department of Regional Health Research, University of Southern Denmark, Odense, Denmark; 3 Centre for Clinical Epidemiology, Odense University Hospital, Odense, Denmark; 4 Research Unit of Clinical Epidemiology, Department of Clinical Research, University of Southern Denmark, Odense, Denmark; 5 Department of Clinical Biochemistry and Pharmacology, Odense University Hospital, Odense, Denmark; 6 Emergency Medicine Research Unit, Hospital Sønderjylland, University Hospital of Southern Denmark, Aabenraa, Denmark; 7 Centre for Prehospital and Emergency Research, Aalborg University Hospital and Institute for Clinical Medicine, Aalborg University, Aalborg, Denmark; 8 Department of Emergency and Trauma Care, Center for Internal Medicine and Emergency Care, Aalborg University Hospital, Aalborg, Denmark; 9 Department of Emergency Medicine, Odense University Hospital, Odense, Denmark

**Keywords:** Prehospital, Lactate, Point-of-care, StatStrip, Portable device

## Abstract

Point-of-care blood lactate is a promising prognostic biomarker of short-term mortality risk. Portable lactate meters need validation in the prehospital setting before widespread implementation and it is unknown whether the mode of sampling (arterial, capillary or venous) matters. This study aims to compare the StatStrip Xpress Lactate Meter’s (SSX) accuracy to a validated blood gas analyser, ABL90 FLEX (ABL90), in arterial samples in the prehospital environment and to determine if lactate levels measured in venous and capillary blood samples are sufficiently accurate compared to arterial lactate levels. Patients with arterial samples drawn by the prehospital anaesthesiologist for any reason were eligible for inclusion. Simultaneously, three blood samples (arterial, capillary and venous) were analysed on SSX and arterial blood on ABL90. Measurements of agreements were evaluated by Lin’s concordance correlations coefficient (CCC) and Bland–Altman Plots. One-hundred-and-eleven patients were included. SSX showed good accuracy compared to ABL90 in arterial samples with a CCC of 0.92 (95% CI 0.90–0.94). Compared to the arterial samples measured on ABL90, venous samples analysed on SSX showed higher agreement than capillary samples analysed on SSX with CCCs of 0.88 (95% CI 0.85–0.91) and 0.79 (95% CI 0.72–0.85), respectively. Bland–Altman plots showed that SSX lactate measurements in arterial, venous and capillary blood samples all had systematically negative biases compared to ABL90. We conclude that the SSX is accurate in our prehospital setting. Venous samples should be preferred over capillary samples, when arterial samples cannot be obtained.

## Introduction

A fast medical assessment is crucial in the treatment of severely injured or acutely ill patients. This assessment includes a focused clinical examination along with measurements of vital values as heart rate, blood pressure, and oxygen saturation. Blood lactate level is often used as a supplement to assess acutely ill or injured patients at the hospital. Measurement of blood lactate levels is an essential diagnostic tool in the intensive care unit (ICU) as well as in the emergency department where it helps to guide patient treatment. Presently, the use of blood lactate measurement is not used routinely in the prehospital environment in the encounter with similar patients [1].

Several studies have reported that blood lactate level may serve as a valid prognostic factor for mortality [2–4]. The blood samples used in these studies were syringes with arterial blood gases (ABG). This is not feasible in most prehospital settings, but new studies emerging on blood lactate measurements show that blood lactate also is a valuable tool for triage in this setting [5, 6]. Most of these studies have reported the use of venous blood drawn from an intravenous line. However, capillary blood is also used in some settings [5–8].

Only a few of the handheld point-of-care (POC) devices measuring blood lactate are approved for medical use. These devices vary in size and differ in their strengths and limitations when used in the field. Their accuracies are tested within laboratories and in hospitals in standardized settings, usually under controlled circumstances including both the preparation of the skin before obtaining the blood sample and the ambient temperature and humidity. Most of these validation studies have shown acceptable results compared to larger—and calibrated—stationary laboratory devices [9–11]. Although in theory very useful in the prehospital setting, the accuracies of the handheld devices are yet to be determined in prehospital settings.

The primary aim of this study was to validate a POC blood lactate measurement device used under prehospital conditions. This was done by evaluating the accuracy of a handheld device measuring blood lactate and compare it to a calibrated mobile device in a prehospital setting.

As arterial blood sampling is generally unfeasible in the prehospital environment, the secondary aim of the study was to investigate whether blood lactate levels measured in capillary and/or venous blood could substitute arterial blood lactate levels to an acceptable extent.

## Method

### Study design

This was a prospective convenience study of the accuracy obtained in prehospital use of a hand-held lactate measurement device. The prehospital setting was the catchment area of the Odense Mobile Emergency Care Unit (MECU), operating in a mixed rural/urban area in Denmark [12]. Since 2013, this MECU has been equipped with an arterial blood gas analyser, the ABL90™ FLEX (Radiometer, Brønshøj, Denmark) [13].

Inclusion criteria:

- The patients were eligible for inclusion in this study if, at any time during their prehospital treatment, the prehospital MECU anaesthesiologist decided to draw an arterial blood gas sample.

Exclusion criteria:

- Patients in whom arterial blood sampling was unfeasible or patients with time-critical illnesses in whom extremely urgent treatment was prioritized.
- Blood samples were excluded from the analyses if they were analysed more than five minutes before or after the reference arterial lactate measurement.

Patient inclusion took place from the 30th of May 2019 until the 31st of January 2020.

### Study approval

The study was regarded as a quality control study by The Regional Committees on Health Research Ethics in Southern Denmark and the need for patients’ consents was waived as stated by the Ethical Committee (Ref. 20192000-54).

### Analysis equipment

Blood lactate levels were measured with StatStrip® Xpress™ Lactate (SSX) (Nova Biomedical, Waltham, Massachusetts, USA) and compared with the results provided by a stationary POC device, ABL90™ FLEX (ABL90) (Radiometer, Brønshøj, Denmark) placed inside the MECU vehicle. The ABL90 used as a reference in this study has been slightly modified. A newly constructed sample inlet was designed by the company Radiometer and the ABL90 was placed in a specially designed cushioned cradle within the MECU vehicle to sustain vibrations and gravitational forces [13]. The SSX was placed in the MECU vehicle’s glove compartment or the storage area between the driver’s and the passenger’s seats to make it immediately accessible.

The SSX requires 0.7 µL blood and has an analysis time of 13 s. Its measurement range is 0.3–20.0 mmol/L and it has an operational temperature span of 15–40 °C. At the time of the study, the device was only approved for medical use in arterial and venous blood samples. The device measures the size of the electrical current produced as a result of the chemical reaction between lactate in the blood sample and the enzyme lactate oxidase in the test strip. The test strips correct for haematocrit, ascorbic acid, uric acid, oxygen, acetaminophen (paracetamol), and bilirubin. In comparison, the ABL90 requires a sample volume of 65 µL and measures the lactate level (along with other parameters) within 35 s. The ABL90 measures lactate levels in whole blood using an amperometric metabolite sensor. The device uses the amount of electrical current flowing through the sensor inside the device to calculate the concentration of lactate being oxidized.

The ABL90 has been a part of the MECU standard equipment since 2013. The device and an additional backup device are subject to regular maintenance and calibration carried out at the Department of Clinical Biochemistry at Odense University Hospital. Analytical performance in terms of analytical coefficient of variation (CV) and imprecision are assured by automated quality management using three different quality control levels analysed automatically every 8th hour and a sensitivity calibration of pCO2, cGlucose, cLactate and the oximetry parameters every 4th hour. Furthermore, a status calibration of all parameters (except the oximetry parameters) is automatically performed prior to any patient measurement, followed by analysis of quality control materials. If Westgard rules are not fulfilled, the automated quality management hindered further analysis until the instrument is recalibrated. The Westgard rules applied are similar to those used for routine blood gas instruments at the laboratory, and accordingly, ABL90 measurements are routinely compared to an in-hospital master device (ABL 837). ABL90’s CVs were 5.1% (4.0 mmol/L), and 5.2% (8.0 mmol/L) at the time of the study. In comparison, the biological variation is 27.2% with a total error of 30.4% according to Ricos et al. [14] Bias was recorded to < 0.2 mmol/L for all levels tested (larger bias has, however, been reported for lactate levels above 15 mmol/L).

The SSX was quality tested in a laboratory before initiation of the study. The quality control was performed with quality solution 1 and 2 along with three discarded whole blood samples containing different lactate concentrations. Quality control solution 1 (QCS1) had a documented lactate concentration of 0.6 mmol/L, and the measurements had to be in the interval 0.3–0.9 mmol/L. Quality control solution 2 (QCS2) had a documented lactate concentration of 6.4 mmol/L, and the measurements had to be in the interval 5.4–7.4 mmol/L. The manufacturer states that typical within-run and day-to-day imprecisions have CVs of 9.1% (0.76 mmol/L), 5.9% (2.16 mmol/L) and 3.4% (10.5 mmol/L).

One investigator tested the SSX once a week with QCS1 and QCS2 during the study period according to the manufacturer’s guidelines. These on-site tests were performed in a large heated garage with a temperature within the SSX limits of 15–40 °C.

### Data collection

Before patient inclusion, all MECU physicians were instructed in the use of the SSX device and blood sample techniques in a 20 min training session. Furthermore, the MECU physicians were provided with additional written material and video documentation about the study details as well as blood sampling techniques. The MECU physicians completed a case report form (CRF) for each patient included in the study. The CRF contained four different lactate values and information about complications, if any, obtaining the blood samples and performing the analyses.

Study patients delivered three blood specimens to produce four lactate measurements. Arterial, capillary and venous blood samples were drawn as close in time as possible. An arterial blood specimen was drawn in a syringe for the ABL90 lactate measurement. The analysis was performed immediately to guide treatment in the clinical setting. The lactate level in this analysis was regarded as the reference lactate level. The remaining arterial blood in the syringe after ABL90 analysis was used for the arterial analysis on SSX. The venous specimen were obtained following intravenous access. A single-use lancet was used to obtain capillary whole blood samples. Before the lancet was used, the skin area was cleaned thoroughly with two alcohol wipes and afterwards dried with sterile gauze. This was done to remove all cutaneous contaminations, in particular drops of sweat, which contains lactate and could lead to erroneous lactate measurements. The first drop of blood was discarded and the second drop of blood was thus used for lactate measurement on the SSX. The MECU anaesthesiologists were instructed to refrain from squeezing the skin as this procedure could lead to erroneous measurements (higher lactate concentrations). Any aberrations to this procedure were specifically recorded in the CRF.

All study information was entered into an Excel sheet and finally transferred to STATA (StataCorp LP, College Station, TX, USA, version 16.0) for statistical analyses.

### Statistics

Minimum sample size was calculated using the statistical software MedCalc®. An expected mean of difference = 0.3 mmol/L and an expected SD of difference = 0.6 was chosen according to the results seen in a previous study [9]. Maximum allowed difference between methods was in this previous study set to 0.3 mmol/L, but the same study also found that the SSX measured systematically lower lactate concentrations compared to the ABL800. Therefore, we allowed a much larger maximum difference of 1.8 mmol/L. A significance level of 0.05 (type I error) and a power of 80% (type II error) was chosen according to international standards. With the mentioned assumptions, a minimum of 83 paired arterial lactate measurements was needed to evaluate the SSX’s precision compared to the ABL90.

To assess the agreements between the lactate measurements performed at the SSX and the measurements from the ABL90, scatter plots with linear regression lines and Lin’s concordance correlation coefficient (CCC) were calculated [15]. Furthermore, Bland–Altman plots were produced to visualize the magnitude and direction of bias [16].

To evaluate the rate of misclassification, the samples were divided into 3 predefined groups according to lactate levels measured by the reference method (ABL90): Group 1 (normal lactate, low risk) < 2.0 mmol/L, group 2 (increased lactate, medium risk) 2.0–3.9 mmol/L, and group 3 (high lactate, high risk) ≥ 4.0 mmol/L. These lactate level groups were selected a priori according to previous studies evaluating lactate levels’ prognostic performance [6, 17].

The SSX’s ability to place the patients in the same risk group as the ABL90 was evaluated for arterial, capillary and venous samples, respectively.

## Results

The in-laboratory quality control of the SSX was performed with ten lactate measurements of every quality solution and whole blood sample. QCS1 and QCS2 showed CV of 0.0% and 1.9%, respectively. Three whole blood samples with mean lactate concentrations of 2.25, 4.80 and 8.05 mmol/L showed CVs of 4.8%, 4.3% and 3.1%, respectively. All five CVs were better than those stated by the manufacturer.

The SSX showed much higher on-site CVs during the 37 study weeks than in-laboratory prior to study initiation. For QCS1, the SSX measured lactate concentrations from 0.5 to 0.9 mmol/L with a mean lactate concentration of 0.70 mmol/L, a standard deviation (SD) of 0.093 and a CV of 13.2%. For QCS2, the SSX measured lactate concentrations from 5.4 to 6.9 mmol/L with a mean lactate concentration of 6.26 mmol/L, a SD of 0.45 and a CV of 7.1%.

**Fig. 1:**
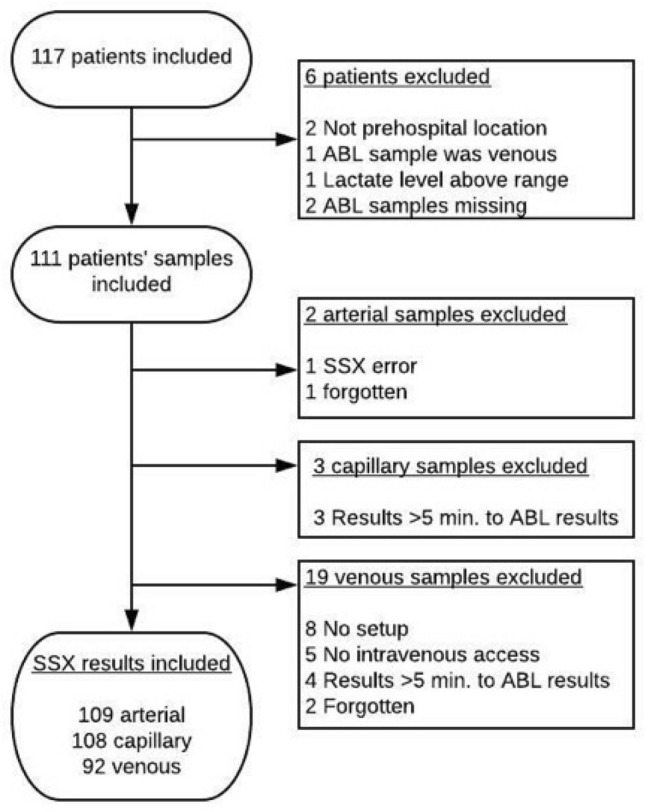
Flowchart of patient inclusion and reason for exclusion

117 patients were included in the study. For flow chart, see Fig. 1. Two patients did not have their blood samples drawn before arrival to the hospital. In one case, the patient’s reference arterial blood lactate level measured on the ABL90 was 26 mmol/L. The three blood samples measured on the SSX device all showed “HI” (= high) indicating measurements above the upper measurement limit of 20.0 mmol/L. One sample, labelled by the MECU physician as “arterial” was, in essence, a venous sample. Altogether, data from 111 patients entered the analyses.

Few arterial and capillary samples were excluded from analyses. However, a total of 19 venous samples were excluded due to causes depicted in Fig. 1. Eight patients were included in the study before we included venous measurements as an outcome. Five patients in whom no venous access could be provided in time for the ABL90 measurements are also missing venous lactate measurements.

In 11 of the 111 included patients, conflicting with the instructions provided to the MECU physicians, the patients’ fingers were squeezed to obtain the necessary amount of capillary blood. See Table 1 for details on the four sample groups.

**Table 1:** Blood lactate measurements divided into sampling sites

Sample site	Number	Median (25–75 percentile)	Range (mmol/l)
Arterial ABL90	111	2.2 (1.3–4.7)	0.4–19.0
Arterial SSX	109	2.0 (1.0–3.7)	0.3–15.1
Venous SSX	92	2.5 (1.4–4.9)	0.3–13.7
Capillary SSX	108	3.1 (1.7–5.0)	0.6–14.9

*SSX* StatStrip Xpress

The SSX device analysed 334 samples in total in which two measurements resulted in an error code. One error was a “Short sample error” and when sufficient blood was placed on a new test strip, the SSX produced a result. By comparison, the ABL90 could not measure lactate in one sample out of the total 115 arterial samples due to a calibration error.

**Fig. 2:**
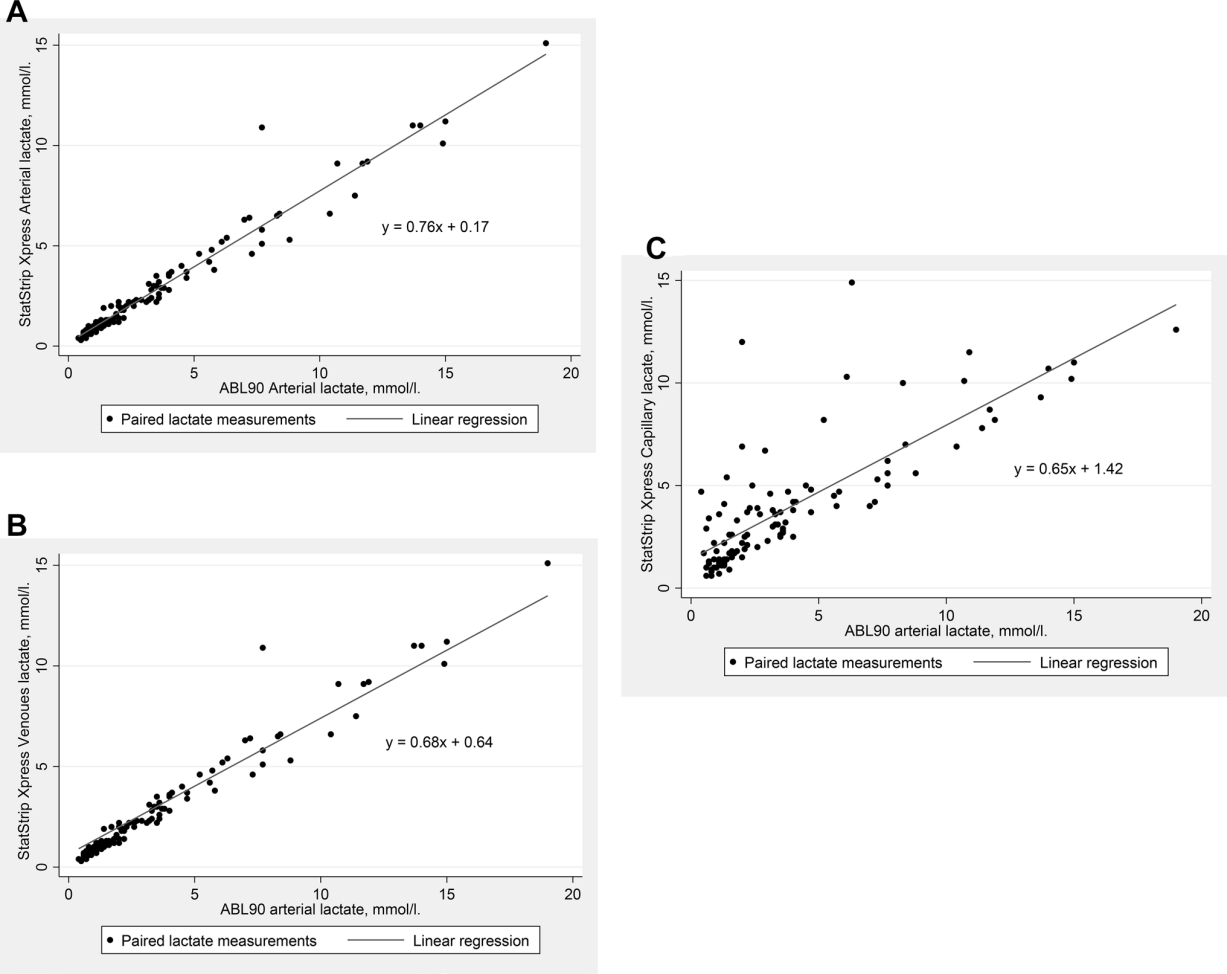
**a** Correlation between the blood lactate levels measured in arterial samples by StatStrip Xpress compared to measurements made by ABL90 in the same arterial samples. **b** Correlation between the blood lactate levels measured in venous samples by StatStrip Xpress compared to measurements made by ABL90 in arterial samples. **c** Correlation between the blood lactate levels measured in capillary samples by StatStrip Xpress compared to measurements made by ABL90 in arterial samples

Figure 2a–c show the linear correlations between the arterial ABL90 measurement and the arterial, venous and capillary measurements performed at the SSX, respectively. Lin’s CCC are 0.92 (95% CI 0.90–0.94) for arterial samples, 0.88 (95% CI 0.85–0.91) for venous samples and 0.79 (95% CI 0.72–0.85) for capillary samples, meaning that the correlations to ABL90’s arterial measurements are very good in the SSX’s arterial measurements, good in the SSX’s venous measurements and acceptable in SSX’s capillary measurements. Figure 2a–c illustrate the same tendencies.

**Fig. 3:**
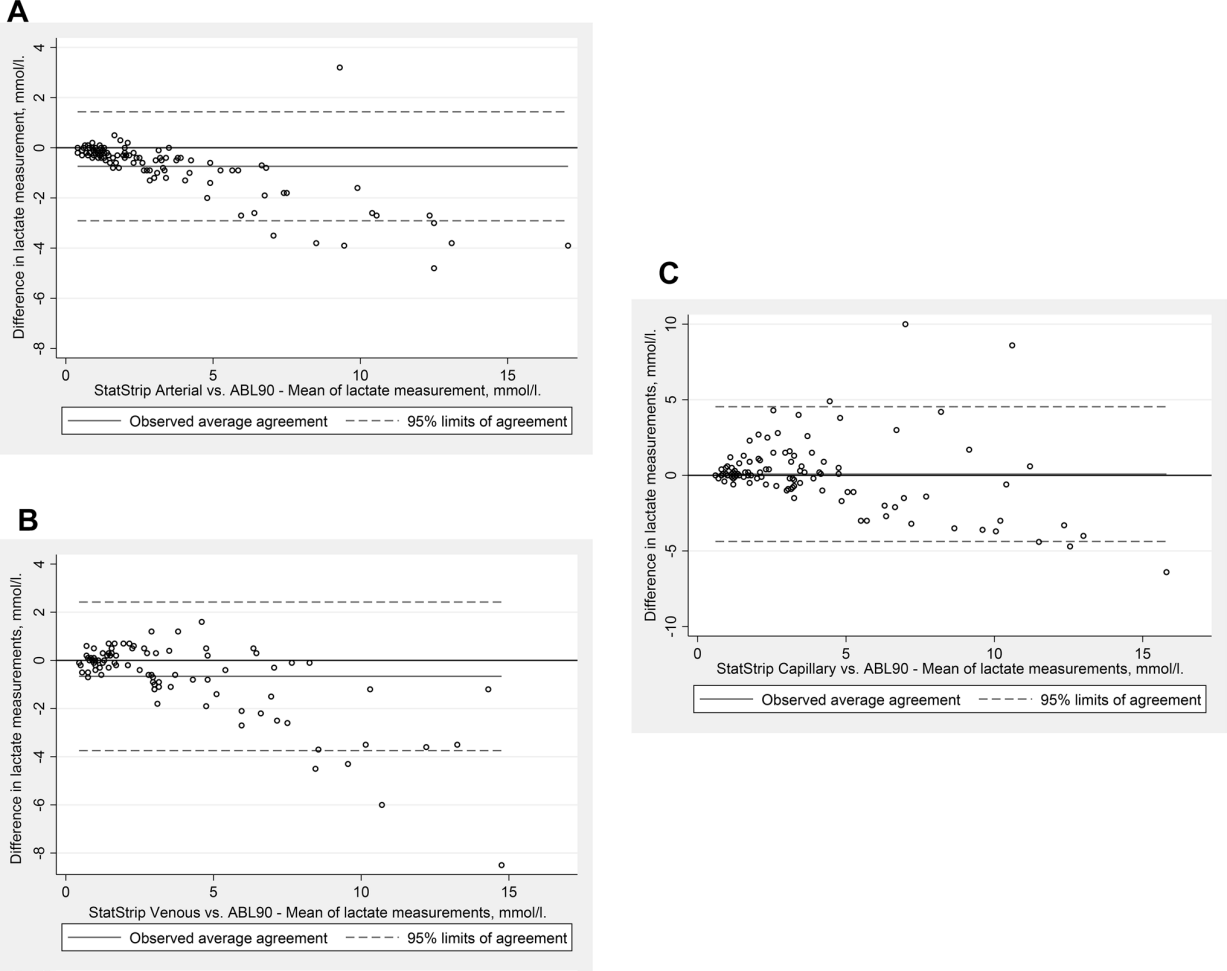
**a** Bland–Altman plot of blood lactate levels in arterial samples measured by StatStrip Xpress compared to measurements made by ABL90 in the same arterial samples. **b** Bland–Altman plot of blood lactate levels in venous samples measured by StatStrip Xpress compared to measurements made by ABL90 in arterial samples. **c** Bland–Altman plot of blood lactate levels in capillary samples measured by StatStrip Xpress compared to measurements made by ABL90 in arterial samples

A Bland–Altman plot of the arterial samples measured on both SSX and ABL90 is shown in Fig. 3a. There was very good correlation between the SSX device’s arterial measurements compared to our reference, the ABL90’s measurements with a mean bias of − 0.74 mmol/L (95% limits of agreement − 2.91–1.43 mmol/L). The SSX showed a high accuracy when measuring low blood lactate levels but increasingly underestimated the true value, the higher the blood lactate levels got. In average, when the ABL90 measured an arterial lactate level of 1.0 mmol/L, the SSX measures a lactate level of 0.76 mmol/L in the same arterial sample. Consequently, this systematically negative bias increased with increasing lactate level.

Figure 3b and c show the Bland–Altman plots of the venous and capillary samples, respectively, measured on the SSX compared to the arterial samples measured on the ABL90. Venous samples had a mean bias of − 0.66 mmol/L (95% limits of agreement − 3.75–2.42 mmol/L.). Capillary samples had a mean bias of 0.08 mmol/L (95% limits of agreement − 4.37–4.54 mmol/L). Both sampling sites showed increasing bias with increasing lactate levels. At low lactate levels, the capillary and especially the venous measurements only showed little absolute difference from the ABL90’s arterial measurements, whereas at high lactate levels the absolute difference became substantial. When comparing Fig. 3a–c, note the differences in ranges on the y-axis.

The number and direction of misclassifications are shown in Table 2.

**Table 2:** Risk group misclassifications

Sample site	ABL90	Arterial SSX	Venous SSX	Capillary SSX
Number total	111	109	92	108
ABL90 RG1	47			
RG1 correct		46 (97.9%)	37 (92.5%)	33 (73.3%)
A higher risk group		1	3	12
ABL90 RG2	31			
RG2 correct		22 (73.3%)	19 (90.5%)	22 (73.3%)
A lower risk group		8	–	2
A higher risk group		–	2	6
ABL90 RG3	33			
RG3 correct		25 (78.1%)	26 (83.9%)	30 (90.1%)
A lower risk group		7	5	3

All lactate measurements were divided into risk groups (RG) with RG1 (normal lactate, low risk) < 2.0 mmol/L, RG2 (increased lactate, medium risk) 2.0–3.9 mmol/L and RG3 (high lactate, high risk) ≥ 4.0 mmol/L. The three different sampling sites (arterial, capillary, and venous) for SSX measurements are separately compared to the measurements performed at the ABL90

*SSX* StatStrip Xpress

## Discussion

### Device accuracy

Our study evaluate the SSX device’s accuracy as good in a prehospital setting compared to the ABL90 in arterial blood samples. The correlation was best at lactate levels lower than 6 mmol/L due to a systematically negative bias. We consider the SSX device as a valuable tool in clinical decision-making in a prehospital setting. Knowledge of a patient’s lactate level at a very early state could lead to better and earlier treatment of time-critical conditions [2]. As a high lactate level indicates a higher risk of mortality [3–7], early prehospital lactate level measurement could improve patient triage in high risk patients.

Several studies have evaluated the SSX’s in-hospital accuracy and found it as accurate as larger stationary laboratory equipment [10, 11]. Colon-Franco et al. analysed discarded blood samples in a laboratory to evaluate the accuracy of the SSX compared to the Radiometer ABL800 FLEX (ABL800) [9]. In accordance with our findings, they found good agreement between the two devices’ measurements and a systematic negative bias on the SSX results compared to the ABL800, especially when the blood lactate level were above 4 mmol/L. Their regression analysis for adult samples showed a slope of 0.80 and a correlation coefficient (r^2^) of 0.98.

Léguillier et al. evaluated the SSX in a more clinically relevant setting in septic patients in the ICU [18]. They found an excellent correlation between the SSX and four central laboratory analysers including the ABL800 using capillary blood the SSX and venous blood for the other analysers. Their regression analysis on SSX vs ABL800 results produced a very fine r^2^ = 0.98. A slope of 0.9 showed a systematically negative bias in SSX measurements.

For clinical purposes, a very high blood lactate level does not have to be entirely accurate, as most health care professional will consider a blood lactate level > 6 mmol/L as very high regardless of the exact concentration. On the contrary, the SSX needs to be precise at the lower lactate levels, as a level of 2.0 mmol/L often, in a clinical context, is used as a cut-off to identify patients who have an increased risk of poor outcome [6, 19]. We evaluated SXX’s ability to make risk stratification in Table 2. Our results show that the SSX only overestimates the risk in one of the 109 arterial samples (lactate levels were 1.7 on ABL90 and 2.0 on SSX, respectively) and about a quarter of the patients with a reference lactate level ≥ 2.0 mmol/L are placed one risk group lower by the SSX compared to the ABL90’s measurements.

The reason for the negative bias is most likely due to the construction of the analytical assays (e.g. difference in antibody/antigen ratio) but could also be related to the distinct difference in amount of test material (0.7 µL vs 65 µL). It is however important to stress that a difference being stable over the measurement range does not have any clinical consequences if a different cut-off or reference interval is applied.

### Comparison of different sampling sites

The second objective of our study was to compare blood lactate levels at different sampling sites as arterial blood sampling is not feasible in many prehospital cases. Our study shows a good correlation between venous lactate levels measured on the SSX and arterial lactate levels measured on the ABL90. Capillary blood lactate levels showed clearly larger bias than venous lactate level when compared to arterial blood lactate measured on ABL90.

We have evaluated the ability to perform a risk stratification based on the two different sampling sites in Table 2. Venous blood samples analysed on the SSX compared to arterial blood samples analysed on the ABL90 showed lower misclassification than arterial samples analysed on the SSX compared to ABL90 analyses. A reason for this could be that blood lactate levels in venous blood are somewhat higher than those of arterial blood in the same patient and this difference reduces the systematically negative bias. Paquet et al. found that venous lactate levels measured in an emergency department were higher than arterial lactate levels, carrying a mean bias of 0.6 mmol/L. The study concluded that it could not be recommended to use the two sampling sites interchangeably [20].

The capillary samples show misclassifications in both directions but these samples mostly overestimates the risk group in spite of the SSX’s general negative bias compared to ABL90’s measurements. Eleven capillary samples were collected while squeezing the patients’ fingers. This, however, was not associated with a higher mean bias or a higher risk of misclassification. Some capillary lactate measurements with very high levels compared to both venous and arterial measurements could have been contaminated with sweat from the patients’ skin due to inadequate disinfection prior to blood sampling.

Another study has evaluated a handheld device, the Accutrend, in the prehospital environment. Stoll et al. compared lactate levels measured prehospitally in venous and capillary blood referred to an in-hospital measurement of venous blood drawn prehospital. The study reported poor overall agreement. The Accutrend showed a positive bias in contrast to the SSX. [8]

### Strengths and limitations

Our study is the first, as we know of, to evaluate the performance of a handheld POC lactate device in a prehospital setting with the reference method also being a prehospital analyser. Our study reflects the accuracy of the device in real life as data were collected by 15 MECU physicians and not just one dedicated investigator. In addition, our study shows that the device is easy to handle with limited training for the personnel in the prehospital environment. Similar device feasibility has been shown for another handheld blood lactate device in a prehospital setting [21]. We consider it a strength of the study that the patient population included in the study to a large extent were seriously ill indicated by the required presence of the MECU and the generally high lactate levels being ≥ 4.0 mmol/L in 28.7% of our patients.

Our study has some limitations when testing the SSX’s accuracy as this is better done in a laboratory not influenced by external conditions (rain, snow and cold temperatures) and operational variations. The reduced possibility to obtain standardised circumstances while obtaining the samples may reduce the accuracy of any point-of-care measurements from levels obtained during controlled conditions. Another limitation of our study is the number of missing data, in particular in the venous lactate measurement group.

We selected the fingers for capillary samples as they are often easily accessible. This choice cause a possible risk of sample contamination. One study suggests the ear lobe as a better alternative when measuring lactate level in hemodynamically compromised patients [22]. Another study concluded that capillary blood from the ear lobe and fingertips could be used for lactate measurements interchangeably, but this study only tested healthy men [23]. Léguillier et al. found excellent agreement between lactate measured in capillary blood samples obtained from fingers and lactate measurements obtained from venous blood samples in septic patients, but blood flows in septic ICU patients are probably not comparable to the blood flows in unselected prehospital patients treated under different weather conditions. In the latter of the two, local hypo perfusion might play an important role in the capillary lactate level.

Finally, it must be mentioned that the quality controls designated the ABL90 FLEX is not optimal in terms of lactate concentrations as it is missing a concentration near or within the clinical relevant area (e.g. 2 mmol/L).

## Conclusion

Our study showed a good correlation between arterial blood lactate levels measured on a handheld device, the StatStrip Xpress, compared to our reference device, ABL90, in the prehospital setting. Our study found the SSX sufficiently sturdy for use in the prehospital setting as the device only produced an error in two out of 334 blood samples analysed. The SSX’s measurements demonstrated a systematically negative bias. Based on our results, we further suggest venous blood samples as a good alternative to arterial samples due to low bias and risk of misclassification. We consider the capillary blood samples as inferior to venous blood samples when attempting to estimate the true arterial lactate levels in our study setting.

## Data Availability

Study data is available by reasonable request by contacting the corresponding author.
